# Manganese and acute paranoid psychosis: a case report

**DOI:** 10.1186/1752-1947-5-146

**Published:** 2011-04-12

**Authors:** Willem M Verhoeven, Jos I Egger, Harold J Kuijpers

**Affiliations:** 1 Vincent van Gogh Institute for Psychiatry, Centre of Excellence for Neuropsychiatry, Venray, The Netherlands; 2 Erasmus University Medical Centre, Department of Psychiatry, Rotterdam, The Netherlands; 3 Radboud University Nijmegen, Donders Centre for Cognition, Nijmegen, The Netherlands; 4 Radboud University Nijmegen, Behavioural Science Institute, Nijmegen, The Netherlands; 5 Vincent van Gogh Institute for Psychiatry, Outpatient Department Deurne, Venray, The Netherlands

## Abstract

**Introduction:**

Manganese regulates many enzymes and is essential for normal development and body function. Chronic manganese intoxication has an insidious and progressive course and usually starts with complaints of headache, fatigue, sleep disturbances, irritability and emotional instability. Later, several organ systems may be affected and, due to neurotoxicity, an atypical parkinsonian syndrome may emerge. With regard to neuropsychiatry, an array of symptoms may develop up to 30 years after intoxication, of which gait and speech abnormalities, cognitive and motor slowing, mood changes and hallucinations are the most common. Psychotic phenomena are rarely reported.

**Case presentation:**

We describe the case of a 49-year-old Caucasian man working as a welder who was referred to our facility for evaluation of acute paranoid psychotic behavior. Our patient's medical history made no mention of any somatic complaints or psychiatric symptoms, and he had been involved in a professional career as a metalworker. On magnetic resonance imaging scanning of his brain, a bilateral hyperdensity of the globus pallidus, suggestive for manganese intoxication, was found. His manganese serum level was 52 to 97 nmol/L (range: 7 to 20 nmol/L). A diagnosis of organic psychotic disorder due to manganese overexposure was made. His psychotic symptoms disappeared within two weeks of treatment with low-dose risperidone. At three months later, serum manganese was decreased to slightly elevated levels and the magnetic resonance imaging T1 signal intensity was reduced. No signs of Parkinsonism were found and a definite diagnosis of manganese-induced apathy syndrome was made.

**Conclusion:**

Although neuropsychiatric and neurological symptoms caused by (chronic) manganese exposure have been reported frequently in the past, in the present day the disorder is rarely diagnosed. In this report we stress that manganese intoxication can still occur, in our case in a confined-space welder, and may present clinically with a paranoid psychotic state that necessitates a rapid diagnostic procedure in order to avoid the permanent structural brain damage that may occur with chronic exposure.

## Introduction

Manganese (Mn) is an essential trace element that regulates and binds to many enzymes throughout the body. In cases of overexposure via inhalation or ingestion, Mn is highly toxic for several organ systems. Mn crosses the blood-brain barrier by the same mechanism that iron does. A peculiar neurological picture similar to Parkinson's shaking palsy due to Mn intoxication was first reported in 1837 [1]. In the first post-war decades of the 20th century, Mn intoxication was documented to occur primarily in miners and welders, manifesting as increased rates of, for example, digestive tract and lung cancer, liver cirrhosis and heart disease. The most common somatic sequelae are hypertension and increased heart rate due to blocking of calcium channels by Mn, elevated cholesterol levels following reduced conversion of cholesterol to bile acids and decreased fertility in men as well as increased fetal abnormalities [2].

With respect to the central nervous system, Mn overexposure initially induces non-specific symptoms such as headache, asthenia, irritability, fatigue, sleep disturbances and emotional instability. Later, a neurodegenerative syndrome with psychiatric symptoms, called manganism, may develop that is characterized by speech, gait and balance problems on the one hand, and obsessive-compulsive behaviors, hostility, mood changes, psychotic experiences such as hallucinations and paranoid ideation, and reduced cognitive flexibility on the other hand [3-5].

In a previous report by Kim *et al.*, an magnetic resonance imaging (MRI) scan of an Mn-overexposed patient showed a regional distribution of high signal intensities in the globus pallidus (100%), midbrain (80%), pituitary gland (43%) and putamen (16%) [6]. The increased signal intensities in the nigrostriatal structures, particularly the globus pallidus, are best demonstrated on T1-weighted images and correlate positively with the Mn concentration in the blood [7,8]. These findings correspond with the neurological syndrome of atypical parkinsonism that comprises, apart from general bradykinesia, rigidity and kinetic tremor, frequent dystonias and a specific cock-walk gait with a tendency to fall forward [9]. In contrast to the findings in patients with Parkinson's disease, positron emission tomography (PET) studies in patients exposed to Mn in general show no dopaminergic abnormalities [10]. Single-photon emission computed tomography (SPECT) images of dopamine transporter (DAT) also demonstrate an overall normal DAT uptake in the striatum [11]. More recent findings in non-human primates suggest that chronic Mn exposure induces a cellular stress response leading to neurodegenerative changes in the frontal cortex that coincide with subtle cognitive deficits [12-14].

In this report, we describe the case of a middle-aged man referred to our out-patient clinic for psychiatric evaluation because of an acute paranoid psychosis.

## Case presentation

Our patient was a 49-year-old Caucasian man who's educational history showed he had completed primary school only. He had been employed in a professional capacity as a metalworker and welder for many years. His somatic history mentioned only slight hypertension, for which he was treated with 25 mg hydrochlorothiazide daily. He was referred for paranoid ideation, thoughts of reference, sleep disturbances and bizarre behaviors. A provisional diagnosis of acute paranoid psychosis was made and our patient was subsequently treated with 2 mg risperidone daily. Within two weeks the psychotic symptoms gradually disappeared and the dose of risperidone was lowered to 1 mg daily. Physical and neurological assessments did not reveal any abnormalities. Routine hematological and biochemical tests, including hemoglobin, lipoprotein profile and fasting glucose, were all within normal ranges. Borrelia and lues serology results were negative. Use of medications including over the counter preparations and illicit drugs were excluded by systematic evaluation.

**Figure 1:**
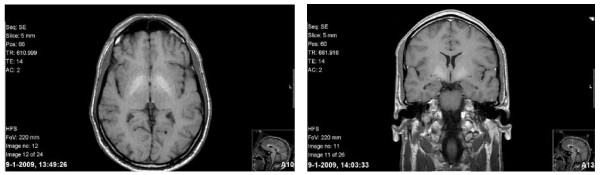
**MRI scan of the brain of our 49-year-old patient exposed to Manganese**. Bilateral high T1 signal intensity in the globus pallidus is shown. Left: transversal view; right: coronal view.

Since there was no personal psychiatric history or family history of psychiatric diseases, a central nervous system disorder was suspected. Subsequently, MRI scanning of the brain was performed that demonstrated high T1 signal intensity of the globus pallidus bilaterally (Figure 1). Analysis of lumbar cerebrospinal fluid showed no abnormalities. Based on the MRI findings, the serum concentration of Mn was measured, which appeared to be increased (52 to 97 nmol/L, reference values: 7 to 20 nmol/L; Laboratories of Clinical Pharmacology, Radboud University Nijmegen, The Netherlands). A definite diagnosis of an organic psychotic disorder due to Mn intoxication was made. As a consequence, DAT-SPECT was performed that showed a normal uptake. Electrocardiography and X-ray of the thorax as well as ultrasonography of the liver displayed no abnormalities.

At three months after the initial referral, a follow-up MRI of the brain again showed enhanced T1 signal intensity in the same areas, but clearly to a lesser extent. The serum Mn concentration had decreased to high normal levels (31 to 36 nmol/L). On neuropsychiatric evaluation, our patient presented with a flat affect and mental dullness. There was a marked discrepancy between his appearance and his stated age. A detailed neurological examination disclosed, apart from a sluggish and indifferent attitude, no abnormalities, especially no signs or symptoms indicative for a parkinsonian syndrome. With respect to psychopathology, using the elements of the Comprehensive Psychiatric Rating Scale his thinking was slow and rigid and, apart from irritability and impulsivity, no formal psychiatric disturbances could be detected. His behavior was dominated by loss of interest in daily activities and social withdrawal as well as a lack of concern about his personal life and intentions. A neuropsychological assessment demonstrated a total IQ of 62 (Kaufman Adult Intelligence Test), which is in agreement with the estimated pre-morbid total IQ of 57 (National Adult Reading Test). There was a slow speed of information processing and a marked cognitive dysfunction with respect to attention, memory (learning and retrieval) and executive functioning (flexibility and shifting).

At follow-up after six months, a further neuropsychiatric examination disclosed findings similar to those obtained previously and treatment with risperidone was discontinued. His wife reported that the changes in his behavior, mood and interests had started insidiously several years before the first psychotic symptoms. A final diagnosis of apathy syndrome caused by chronic overexposure to Mn was formulated. Subsequently, psychosocial supportive therapy was given and our patient will be re-examined twice yearly in order to monitor the potential development of parkinsonian symptoms.

## Discussion

In our 49-year-old patient, a bilaterally increased signal intensity in the globus pallidus on T1-weighted MRI was found. It appeared to be caused by overexposure to Mn as demonstrated by an elevated serum concentration of Mn. Therefore, a diagnosis of organic psychotic disorder/apathy syndrome due to Mn intoxication was made. This diagnosis was supported by the decrease of serum levels of Mn and the reduction of bilateral globus pallidus T1 signal intensities over a period of three months. Extensive investigation of our patient's environment revealed no potential sources for Mn exposure other than occupational exposure.

For several decades it has been known that once the neuropathology is clearly expressed at the clinical level, Mn-caused damage to the central nervous system is essentially irreversible and often evolutive [15]. Eventually, an atypical parkinsonian syndrome may emerge that is characterized by gait and balance problems, dysarthria and absence of rest tremor, and points at a striatal-pallidal degeneration [3]. Whether recurrent low-dose occupational exposure to Mn causes early brain damage is still controversial [16].

Several studies have replicated the observation that the nigrostriatal dopaminergic system is essentially intact in manganism, which has become evident from normal PET and DAT-SPECT findings. Thus, in cases of high T1 signal in patients with a history of Mn exposure in whom a normal PET/SPECT uptake is found, a diagnosis of Mn-induced parkinsonism is justified. If PET/SPECT uptake is reduced and MRI shows no abnormalities, a diagnosis of idiopathic Parkinson's disease should be made [11].

Although the results from a few older reports are suggestive for some efficacy of treatment with L-3,4-dihydroxyphenylalanine (L-dopa) or 5-hydroxytryptophan in Mn-induced parkinsonism, no effect could be demonstrated in the only placebo-controlled study [17]. This unresponsiveness to (L-dopa) is most likely related to the difference in the pathophysiology between idiopathic and Mn-induced parkinsonism. It has been repeatedly shown that Mn accumulation in the brain is not associated with degeneration of nigrostriatal dopaminergic neurons [18].

With respect to possible ways to reduce Mn accumulation in brain structures in order to prevent neurological damage, chelating agents such as ethylenediaminetetra-acetic acid (EDTA), have been reported to possibly generate some positive effects in the early stages of poisoning, although sometimes only temporarily. In case of structural brain damage, however, chelating therapy cannot be expected to bring about any improvement [19]. In the latter condition, experimental treatment with para-aminosalicylic acid (PAS), originally used for the treatment of tuberculosis, has been applied with promising alleviation of neurological symptoms [20].

With regard to the neuropsychiatric and psychological effects of Mn overexposure, several domains of dysfunction can be delineated such as: emotional instability with irritability, anxiety, impulsivity and hostility; psychotic symptoms such as paranoid ideation and hallucinations; compulsive behaviors; and cognitive dysfunctions, particularly slowing of information processing, reduced mental flexibility and impaired attention and learning [5]. For these areas of dysfunction, no specific psychopharmacological strategies can be recommended other than the regular treatment modalities [4,9].

## Conclusion

To the best of our knowledge, this is the first report of Mn-induced paranoid psychosis due to confined-space welding. The occurrence of subacute psychotic symptoms in the absence of a psychiatric history may point towards occupational diseases that in the modern world are now rare. In the case of Mn exposure, treatment with EDTA may be effective in the early stages. Later, however, when the neurodegenerative parkinsonian syndrome has become irreversible and sometimes progressive without any further Mn exposure, no therapeutic options remain. Therefore, prevention of Mn intoxication should be the primary target.

## Consent

Written informed consent was obtained from the patient for publication of this case report and any accompanying images. A copy of the written consent is available for review by the Editor-in-Chief of this journal.

## Competing interests

The authors declare that they have no competing interests.

## Authors' contributions

WV and JE guided the investigation, performed neuropsychiatric and neuropsychological assessment and wrote the manuscript HK was responsible for the clinical care and the primary analysis of data from our patient. All authors read and approved the final manuscript.

## References

[B1] Lee JW Manganese intoxication Arch Neurol 2000 57 597 599 10.1001/archneur.57.4.597 10768639

[B2] Crossgrove J Zheng W Manganese toxicity upon overexposure NMR Biomed 2004 17 544 553 10.1002/nbm.931 15617053 PMC3980863

[B3] Jankovic J Searching for a relationship between manganese and welding and Parkinson's disease Neurology 2005 64 2021 2028 10.1212/01.WNL.0000166916.40902.63 15985567

[B4] Bouchard M Mergler D Baldwin M Panisset M Roels HA Neuropsychiatric symptoms and past manganese exposure in a ferro-alloy plant Neurotoxicology 2007 28 290 297 10.1016/j.neuro.2006.08.002 16962176

[B5] Bowler RM Roels HA Nakagawa S Drezgic M Diamond E Park R Koller W Bowler RP Mergler D Bouchard M Smith D Gwiazda R Doty RL Dose-effect relationships between manganese exposure and neurological, neuropsychological and pulmonary function in confined space bridge welders Occup Environ Med 2007 64 167 177 10.1136/oem.2006.028761 17018581 PMC2092523

[B6] Kim Y Kim KS Yang JS Park IJ Kim E Jin Y Kwon KR Chang KH Kim JW Park SH Lim HS Cheong HK Shin YC Park J Moon Y Increase in signal intensities on T1-weighted magnetic resonance images in asymptomatic manganese-exposed welders Neurotoxicology 1999 20 901 908 10693971

[B7] Park NH Park JK Choi Y Yoo CI Lee CR Lee H Kim HK Kim SR Jeong TH Park J Yoon CS Kim Y Whole blood manganese correlates with high signal intensities on T1-weighted MRI in patients with liver cirrhosis Neurotoxicology 2003 24 909 915 10.1016/S0161-813X(03)00111-6 14637385

[B8] Shin YC Kim E Cheong HK Cho S Sakong J Kim KS Yang JS Jin YW Kang SK Kim Y High signal intensity on magnetic resonance imaging as a predictor of neurobehavioral performance of workers exposed to manganese Neurotoxicology 2007 18 257 262 10.1016/j.neuro.2006.03.014 16647136

[B9] Bowler RM Koller W Schulz PE Parkinsonism due to manganism in a welder: neurological and neuropsychological sequelae Neurotoxicology 2006 27 327 332 10.1016/j.neuro.2005.10.011 16457889

[B10] Cersosimo MG Koller WC The diagnosis of manganese-induced parkinsonism Neurotoxicology 2006 27 340 346 10.1016/j.neuro.2005.10.006 16325915

[B11] Kim Y Neuroimaging in manganism Neurotoxicology 2006 27 369 372 10.1016/j.neuro.2005.12.002 16442160

[B12] Guilarte TR Burton NC McGlothan JL Verina T Zhou Y Alexander M Pham L Griswold M Wong DF Syversen T Schneider JS Impairment of nigrostriatal dopamine neurotransmission by manganese is mediated by pre-synaptic mechanism(s): implications to manganese-induced parkinsonism J Neurochem 2008 107 1236 1247 10.1111/j.1471-4159.2008.05695.x 18808452 PMC3675778

[B13] Guilarte TR Burton NC Verina T Prabhu VV Becker KG Syversen T Schneider JS Increased APLP1 expression and neurodegeneration in the frontal cortex of manganese-exposed non-human primates J Neurochem 2008 105 1948 1959 10.1111/j.1471-4159.2008.05295.x 18284614 PMC4335763

[B14] Schneider JS Decamp E Clark K Bouquio C Syversen T Guilarte TR Effects of chronic manganese exposure on working memory in non-human primates Brain Res 2009 1258 86 95 10.1016/j.brainres.2008.12.035 19133246 PMC2659542

[B15] Roels HA Ortage Esleva MI Ceulemans E Robert A Lison D Prospective study on the reversibility of neurobehavioral effects in workers exposed to manganese dioxide Neurotoxicology 1999 20 255 272 10385889

[B16] Greiffenstein MF Lees-Haley PR Neuropsychological correlates of manganese exposure: a meta-analysis J Clin Exp Neuropsychol 2007 29 113 126 10.1080/13803390600781105 17365247

[B17] Koller WC Lyons KE Truly W Effect of levodopa treatment for parkinsonism in welders Neurology 2004 62 730 733 15007122 10.1212/01.wnl.0000113726.34734.15

[B18] Guilarte TR Manganese and Parkinson' s disease: a critical review and new findings Environ Health Perspect 2010 118 1071 1080 10.1289/ehp.0901748 20403794 PMC2920085

[B19] Blanuša M Varnai VD Piasek M Kostial K Chelators as antidotes of metal toxicity: therapeutic and experimental aspects Curr Med Chem 2005 12 2771 2794 16305472 10.2174/092986705774462987

[B20] Shuqin K Haisang D Peiry X Wanda H A report of two cases of chronic serious manganese poisoning treated with sodium para-aminosalicylic acid Br J Ind Med 1992 49 66 69 1733459 10.1136/oem.49.1.66 PMC1039237

